# Within-Host Evolution of *Staphylococcus aureus* during Asymptomatic Carriage

**DOI:** 10.1371/journal.pone.0061319

**Published:** 2013-05-01

**Authors:** Tanya Golubchik, Elizabeth M. Batty, Ruth R. Miller, Helen Farr, Bernadette C. Young, Hanna Larner-Svensson, Rowena Fung, Heather Godwin, Kyle Knox, Antonina Votintseva, Richard G. Everitt, Teresa Street, Madeleine Cule, Camilla L. C. Ip, Xavier Didelot, Timothy E. A. Peto, Rosalind M. Harding, Daniel J. Wilson, Derrick W. Crook, Rory Bowden

**Affiliations:** 1 Department of Statistics, University of Oxford, Oxford, Oxfordshire, United Kingdom; 2 Experimental Medicine Division, Nuffield Department of Clinical Medicine, University of Oxford, Oxford, Oxfordshire, United Kingdom; 3 National Institute for Health Research Oxford Biomedical Research Centre, Oxford, Oxfordshire, United Kingdom; 4 Wellcome Trust Centre for Human Genetics, University of Oxford, Oxford, Oxfordshire, United Kingdom; 5 Oxford University Hospitals National Health Service Trust, Oxford, Oxfordshire, United Kingdom; 6 Department of Primary Care Health Sciences, University of Oxford, Oxford, Oxfordshire, United Kingdom; 7 Department of Zoology, University of Oxford, Oxford, Oxfordshire, United Kingdom; Cairo University, Egypt

## Abstract

**Background:**

*Staphylococcus aureus* is a major cause of healthcare associated mortality, but like many important bacterial pathogens, it is a common constituent of the normal human body flora. Around a third of healthy adults are carriers. Recent evidence suggests that evolution of *S. aureus* during nasal carriage may be associated with progression to invasive disease. However, a more detailed understanding of within-host evolution under natural conditions is required to appreciate the evolutionary and mechanistic reasons why commensal bacteria such as *S. aureus* cause disease. Therefore we examined in detail the evolutionary dynamics of normal, asymptomatic carriage. Sequencing a total of 131 genomes across 13 singly colonized hosts using the Illumina platform, we investigated diversity, selection, population dynamics and transmission during the short-term evolution of *S. aureus*.

**Principal Findings:**

We characterized the processes by which the raw material for evolution is generated: micro-mutation (point mutation and small insertions/deletions), macro-mutation (large insertions/deletions) and the loss or acquisition of mobile elements (plasmids and bacteriophages). Through an analysis of synonymous, non-synonymous and intergenic mutations we discovered a fitness landscape dominated by purifying selection, with rare examples of adaptive change in genes encoding surface-anchored proteins and an enterotoxin. We found evidence for dramatic, hundred-fold fluctuations in the size of the within-host population over time, which we related to the cycle of colonization and clearance. Using a newly-developed population genetics approach to detect recent transmission among hosts, we revealed evidence for recent transmission between some of our subjects, including a husband and wife both carrying populations of methicillin-resistant *S. aureus* (MRSA).

**Significance:**

This investigation begins to paint a picture of the within-host evolution of an important bacterial pathogen during its prevailing natural state, asymptomatic carriage. These results also have wider significance as a benchmark for future systematic studies of evolution during invasive *S. aureus* disease.

## Introduction

Many bacterial pathogens, *Staphylococcus aureus* included, pose an evolutionary puzzle. Despite the heavy burden of disease they impose throughout the world, the prevalence of asymptomatic carriage dwarfs the incidence of disease [1]–[3]. Around a third of healthy adults carry *S. aureus* nasally [1]. In comparison, the yearly incidence in the United States of mortality from *S. aureus* diseases including septicemia, endocarditis and toxic shock syndrome is around 1 per 100,000 people [3]. In the lifecycle of bacteria such as *S. aureus*, disease is therefore an infrequent event, and it is supposed that the bulk of transmission occurs between healthy carriers. This stands in contrast to viruses, the other major group of human pathogens, in whom disease is often the outward manifestation of an obligate part of the cycle of transmission [3].

In consequence, commonly carried bacteria have been labeled accidental pathogens (e.g. [4], [5]), implying that pathogenesis is an evolutionary dead end for the bacteria, with limited significance for transmission. Yet this story is too simple. The armory of virulence factors possessed by *S. aureus*, such as enterotoxins, fibronectin binding protein and gamma hemolysin [6], demonstrates that the species is equipped, at the least, to cause disease facultatively. The rise of *S. aureus* resistant to antibiotics including penicillin and methicillin [7] provides further evidence of an advantage to survival within, and transmission from, sick patients.

Of the factors that affect the balance between commensalism and invasive disease, something is known. Age, sex and underlying health conditions are risk factors for disease, as is ethnicity [8]–[11], suggesting a likely role for host genetics (see also [12]). Bacterial genetics, specifically the presence of key virulence factors, has been shown to be important [6], although no consistent association between virulence and evolutionary lineage has been demonstrated [13], [14]. Carriage is well established as a risk factor for *S. aureus* disease. For example in one study, concomitantly carried *S. aureus* were indistinguishable from invasive bacteria in 82% of cases on the basis of pulsed field gel electrophoresis [15].

A potentially important factor tipping the balance between carriage and disease is evolution of the bacteria within the host. Yet very little is known about within-host evolution in bacteria compared to viruses, where the subject has been the focus of intense research for more than a decade (see e.g. [16] for a review). This is mainly for practical reasons: bacteria have much larger genomes and lower mutation rates [17], resulting in sparse genetic variation in many of the most virulent bacterial pathogens [18], [19]. But recent advances in whole-genome sequencing have made it possible, for the first time, to study bacterial evolution *in vivo* [20], revealing detectable evolution and adaptation on timescales of just a few months (e.g. [21]–[28]). In recent work, we used whole-genome sequencing to investigate the evolution of *S. aureus* during progression from prolonged asymptomatic carriage to a fatal bloodstream infection in a single carrier [29]. Our results showed that bacterial evolution within the host, in particular the substitution of knock-out mutations induced by premature stop codons, was associated with the transition to invasive disease.

Systematic studies are now required to build a detailed understanding of the nature of within-host bacterial evolution during normal carriage and the role it plays in pathogenesis. The aim of this study was to investigate the evolution of *S. aureus* under the prevailing natural conditions, *i.e.* asymptomatic carriage, in order to provide biological insight and facilitate future comparisons to cases of invasive disease. We investigated diversity, natural selection and population dynamics within asymptomatic, singly colonized carriers. Using our results, we demonstrated one of the practical applications of this knowledge by assessing the evidence for recent transmission between our study participants via a newly developed population genetics model.

## Results

### Microvariation is a common feature of nasal carriage

We detected nasal carriage of *S. aureus* in 360 out of 1,123 asymptomatic adults recruited from general medical practices in Oxfordshire, UK. Of those, we selected for further investigation 13 individuals (participants A–M) who carried the common, hospital-associated clonal complex (CC) 22 and CC 30 strains. Carriers showing evidence for mixed colonization on the basis of *spa* typing (a standard molecular typing method [30]), were excluded, and we intentionally over-represented MRSA by including five of the nine MRSA-carrying individuals in the study in order to investigate any difference between carriage of methicillin-sensitive and resistant *S. aureus*. We sequenced the genomes of between eight and twelve bacterial colonies from a single swab sample from each host (131 genomes in total) using the Illumina GAIIx platform (Illumina, San Diego, USA).

**Figure 1:**
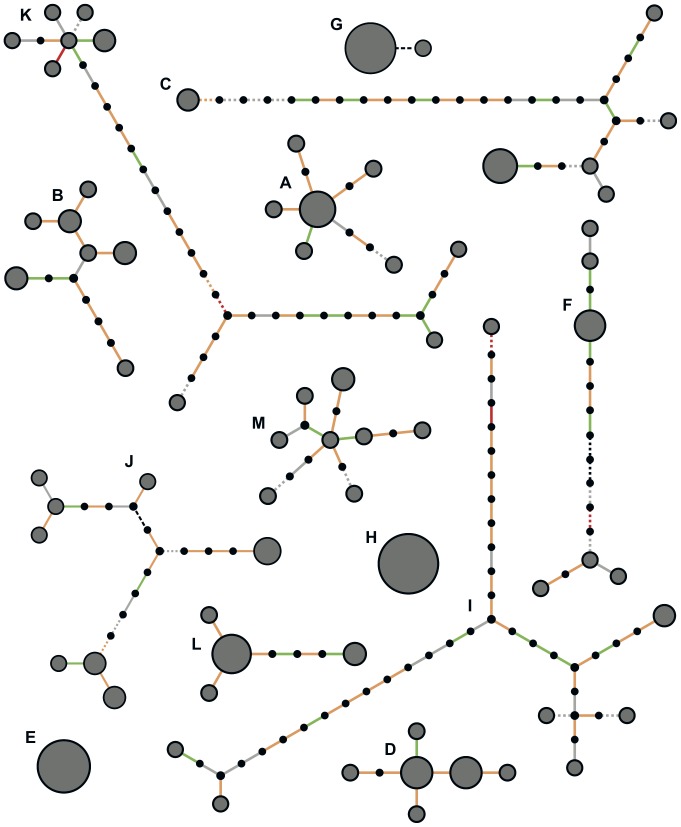
Genomic diversity of *Staphylococcus aureus* in 13 singly-colonized nasal carriers. For each carriage study participant (A–M) a representation of the maximum likelihood tree is shown relating all colonies isolated and sequenced from that host. Gray circles represent observed genotypes, where area is proportional to sample frequency, and small black circles represent hypothetical intermediate genotypes. Edges (branches) represent mutations, color-coded as follows: synonymous (green), non-synonymous (orange), premature stop (red), non-coding (grey), structural variant (black). Solid edges represent SNPs and dashed edges represent indels. The ordering of mutations along a branch is arbitrary.

We found that microvariation – sparse genetic variation in the form of single nucleotide polymorphisms (SNPs) and short insertions/deletions (indels) – was common. Using a combination of reference-based mapping and *de novo* assembly of the genome, we detected microvariation in all but three nasal carriers, comprising a total of 162 unique SNPs and 22 short indels (Table 1). An exhaustive list of the variants detected in participants A–M is provided in Table S1. The number of SNPs per host ranged from six to 40 and the number of short indels from one to six. No variation was detected in the MLST loci, targeted by conventional sequence typing [31]. We found no evidence for within-host recombination using a test based on the relationship between physical distance and linkage disequilibrium (as measured by the *r* ^2^ statistic; see Table S2) [32]–[37]. Indeed, there was no evidence for homoplasy within individual hosts based on the four gamete test [38]. Therefore we used standard tree-building methods to visualize the evolutionary relationships within hosts (Figure 1).

**Table 1:** Genomic diversity in asymptomatically carried nasal populations of *Staphylococcus aureus*.

							Single nucleotide polymorphisms						Short insertions/deletions			
ID	Spa type	MLST	Clonal complex	MRSA	AB use a	Sample size	Syn	Non-syn	Stop	Non-CDS	Total SNP	π ^b^	Frame-shift	Stop	Non-CDS	Total Indel
A	t005	ST22	CC22	–	–	10	1	6	–	1	8	1.60	–	–	1	1
B	t005	ST2257^c^	CC22	–	★	10	2	8	–	1	11	3.27	–	–	–	–
C	t006	ST22	CC22	–	–	11	7	13	–	4	24	7.89	1	–	5	6
D	t032	ST22	CC22	MRSA	–	12	1	5	–	–	6	1.36	–	–	–	–
E	t032	ST22	CC22	MRSA	–	8	–	–	–	–	–	0.00	–	–	–	–
F	t032	ST22	CC22	MRSA	★	9	4	4	–	2	10	3.67	–	1	2	3
G	t032	ST22	CC22	MRSA	★★	11	–	–	–	–	–	0.00	–	–	–	–
H	t012	ST30	CC30	–	–	11	–	–	–	–	–	0.00	–	–	–	–
I	t012	ST30	CC30	–	–	8	7	22	1	10	40	14.75	–	1	2	3
J	t012	ST30	CC30	–	–	12	3	9	–	3	15	5.50	1	–	2	3
K	t012	ST30	CC30	–	–	10	8	16	1	6	31	10.53	1	1	2	4
L	t012	ST30	CC30	–	–	10	2	4	–	–	6	1.82	–	–	–	–
M	t012	ST36	CC30	MRSA	★★	9	2	7	–	2	11	3.11	–	–	2	2
Total						131	37	94	2	29	162		3	3	16	22

a Recent antibiotic use: ★ amoxicillin, ★★ antibiotic with expected anti-staphylococcal activity (trimethoprim or ciprofloxacin). ^b^Average SNP divergence between colonies. ^c^Single-locus variant of ST22.

MLST: multilocus sequence type, MRSA: methicillin resistant *Staphylococcus aureus*, syn: synonymous, stop: premature stop codon, CDS: coding sequence, SNP: single nucleotide polymorphism, indel: insertion/deletion, ST: sequence type, CC: clonal complex.

The distributions of SNP divergence within and between hosts did not overlap, supporting the view that each was a carriage population founded by a single colonization event. The mean SNP divergence between two colonies sampled within the same host was *π* = 4.12 per genome, with a maximum of 26 (participant I). We recorded the recent usage of antibiotics, including those expected to have anti-staphylococcal activity (Table 1). Although recent users had among the lowest diversity of nasal carriage populations, we did not detect a statistically significant effect. Similarly, we observed that MRSA carriage populations showed lower diversity on average, but the difference was not statistically significant.

**Figure 2:**
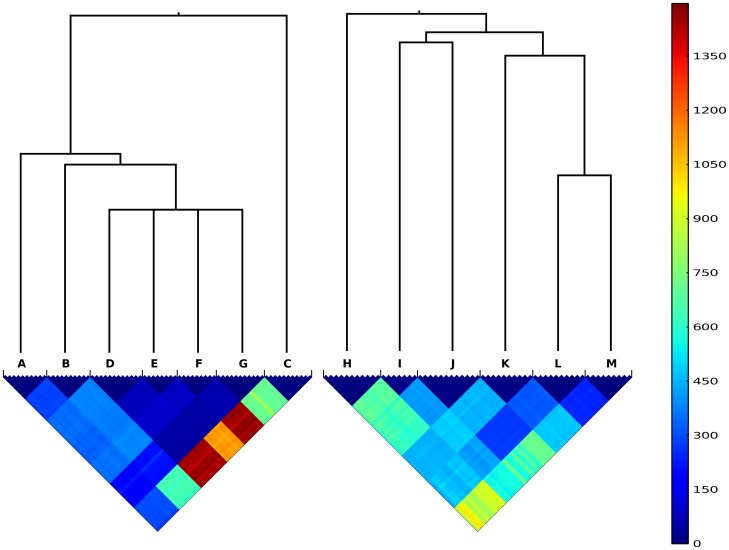
Diversity of *S. aureus* within and between hosts. Separately for CC22 genomes (A–G) and CC30 genomes (H–M), the clonal frame consensus tree representing the relationships between hosts is shown above a heat map indicating the number of SNP differences between pairs of genomes isolated from the same and different hosts. In the key, cooler colors (closer to blue) represent less divergence and hotter colors (closer to red) represent greater divergence.

The mean SNP divergence between colonies sampled from different hosts was 457 within CC22 (range 44–1334), 396 within CC30 (range 211–786) and 22,738 between CC22 and CC30 (range 9,001–33,633), based on mapping to the MRSA252 reference genome [39]. There was evidence of homoplasy between hosts in CC30 and evidence of recombination between hosts in both CC22 and CC30 (Table S2). Consequently, we used ClonalFrame [40] to reconstruct ancestral relationships between hosts, separately for CC22 and CC30 (Figure 2).

### Mobile elements generate within-host structural variation

**Figure 3:**
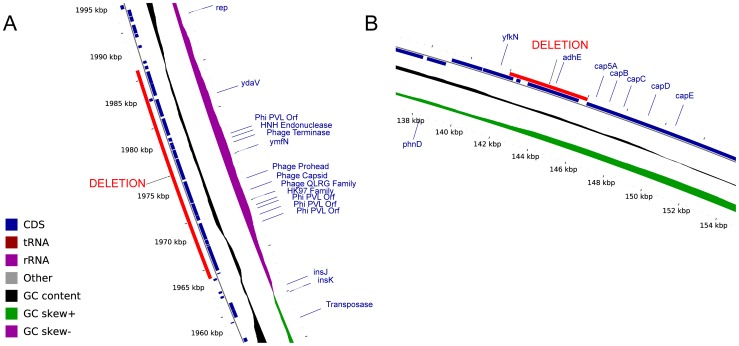
Examples of large insertion-deletion polymorphisms within single hosts. A) 23.9 kb deletion of a Panton-Valentine leukocidin-encoding prophage in four colonies isolated from participant J (contig C618:c65). B) 3.5 kb indel knocking out *adhE* in three colonies isolated from participant F (contig C608:c44). In both panels, the deleted region is indicated in red. The presence of coding sequences (CDS, dark blue), tRNA (dark red), rRNA (purple) and other features (gray) are indicated by filled rectangles. Sliding windows are shown indicating GC content (black), and positive (green) or negative (purple) GC skew. Positions are indicated relative to the concatenated Velvet assemblies of the host-specific reference genomes. Figures extracted from circular chromosome plot generated using CGView [81].

We found evidence of structural variation within some nasal carriage populations that was associated with bacteriophages, and evidence of variation in the presence of plasmids (Table 2). We detected large deletions by mapping the Illumina reads from each colony to the contigs of the host-specific reference assembly. Our criterion for detection was the complete absence of mapped reads for more than 500 bp per kilobase. We used Mauve to validate the deleted regions by aligning the contigs of the genomes concerned [41], [42]. In participant J we detected a large deletion in four of the 12 colonies (colonies C619, C620, C622 and C624) that spanned two contigs (contigs c65 and c80 in the host-specific reference genome C618). We found that these contigs, together comprising 26.1 kb, exhibited homology to *Staphylococcus* phages on the basis of BLAST matches [43]. These phages, known as φPVL [44], contained Panton-Valentine leukocidin (PVL), a cytotoxin that forms pores in the membranes of infected host cells and which is a recognized virulence factor in *S. aureus* [45], [46] (Figure 3A).

**Table 2:** Large structural variation within hosts.

ID	Colonies affected	Event relative to reference	Contigs a	Length (kb)	BLAST hits
F	C611, C612, C615	Deletion	C608:c44	3.5	Aldehyde-alcohol dehydrogenase
		Insertion	C611:c19	2.5	Plasmid pT48 (2.5 kb, M19652.1)
G	C433	Insertion	C433:c20	23.5	*Staphylococcus* phage P954 (40.7 kb, GQ398772.1)
			C433:c43	8.3	*Staphylococcus* phage PVL proviral DNA (41.4 kb, AB009866.2)
			C433:c21	4.0	*Staphylococcus* phage phiNM3 (44.0 kb DQ530361.1)
			C433:c11	3.3	*Staphylococcus* phage P954 (40.7 kb, GQ398772.1)
			C433:c24	2.1	*Staphylococcus aureus* phage phi 13 (42.7 kb, AF424783.1)
J	C619, C620, C622, C624	Deletion	C618:c65	23.9	*Staphylococcus* phage phiPVL108 (44.9 kb, AB243556.1)
			C618:c80	2.2	*Staphylococcus* phage PVL proviral DNA (41.4 kb, AB009866.2)

a Underscoring indicates contigs that were present in the host-specific reference.

We detected large insertions by *de novo* assembly of Illumina reads that did not map to the host-specific reference genome. For detection, we required that the length of the assembled contigs exceed one kilobase, and we used Mauve to validate the insertions. In participant G we detected a large insertion spanning five contigs, with a combined sequence length of 41.2 kb in one out of 11 colonies (contigs c11, c20, c21, c24 and c43 in genome C433). These contigs showed homology to phages φPVL and φNM3 among others (Table 2), the latter present in the Newman reference genome and known to carry virulence factors including enterotoxin A and gamma hemolysin [47], [48].

Although we distinguished insertions from deletions on the basis of comparison to the host-specific reference genome, this nomenclature did not necessarily correspond to the true direction of the event (loss or acquisition). For example, in participant J the phage that was deleted in four of the 12 genomes is not present in the CC30 reference genome (MRSA252). This suggests that the presence of the phage in eight of the colonies, including the host-specific reference genome, may have been the derived state, implying that the phage was ancestrally absent and subsequently acquired rather than lost. Conversely, in participant G the phage that was inserted in one of the 11 genomes did show homology to the CC22 reference genome (EMRSA15). This suggests that the absence of the phage in ten of the colonies, including the host-specific reference genome, could have been the derived state, so that the phage was ancestrally present and subsequently lost rather than acquired.

In participant F however, we detected both a large deletion and a large insertion event. In three out of 9 colonies (genomes C611, C612 and C615), we detected the deletion of a 3.5 kb contig (c44 in the host-specific reference genome C608) containing *adhE*, a gene encoding alcohol-aldehyde dehydrogenase (Figure 3B). In the same three colonies, we detected the insertion of a 2.5 kb contig 99.5% similar to the complete genome of the pT48 plasmid [49] (c19 in genome C611). This plasmid encodes inducible resistance to macrolide-lincosamide-streptogramin B (MLS) antibiotics [49]. The depth of coverage of reads mapping to this contig was around twice the genome average, suggesting the plasmid was present in multiple copies. We postulate that the deletion of *adhE* and the insertion of plasmid pT48 represent two separate events (one loss, one acquisition) on the same branch of the evolutionary tree (Figure 1F), demonstrating that both *de novo* acquisition and loss events can be observed *in vivo*.

### Purifying selection dominates the within-host fitness landscape

**Figure 4:**
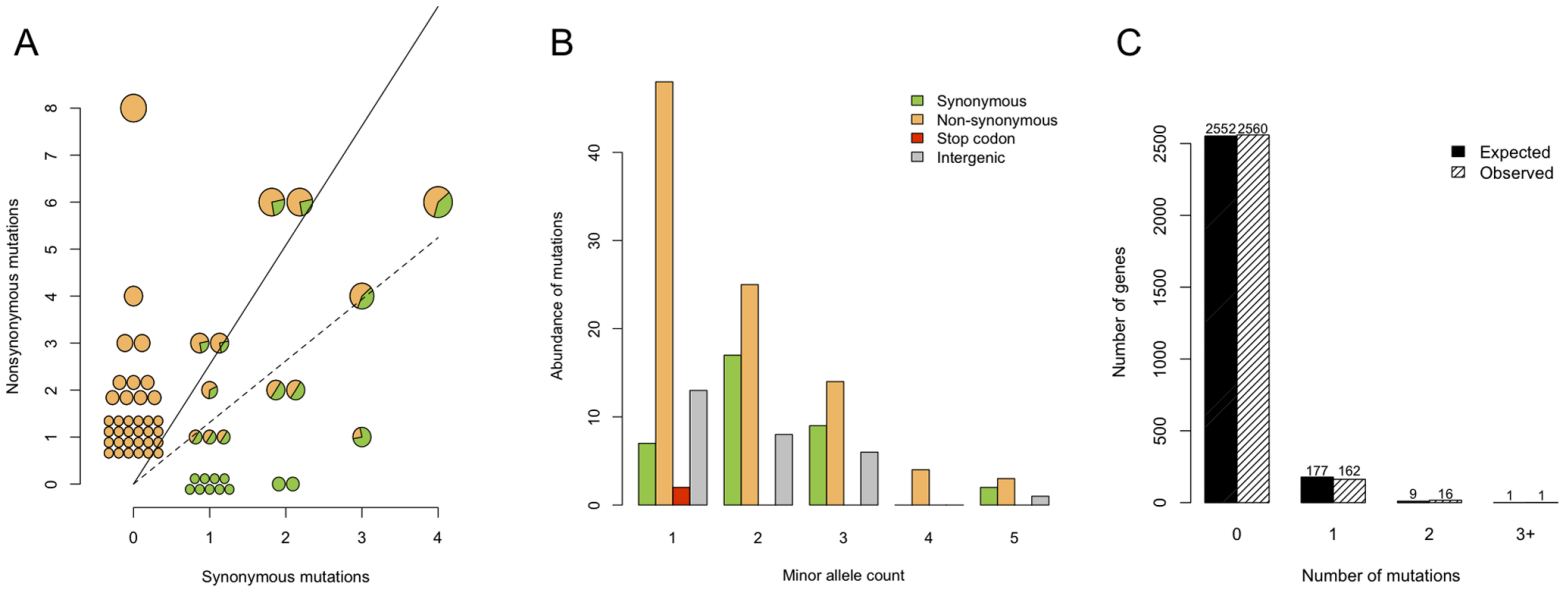
Evidence for natural selection on the *Staphylococcus aureus* genome during asymptomatic carriage. A) The relative number of synonymous versus non-synonymous SNPs on all branches of the within-host genealogies relating colonies sampled from hosts A-M. Each pie represents a branch in Figure 1, divided into segments according to the proportion of synonymous (green) and non-synonymous (orange) mutations on that branch. The area of the pie is proportional to the number of synonymous and non-synonymous mutations on that branch. The solid line is the uncorrected *dN*/*dS* ratio estimated from SNPs within hosts, which was significantly greater than the uncorrected *dN*/*dS* ratio estimated from SNPs between hosts (dashed line, McDonald-Kreitman test *p*<0.001). B) The sample frequency of SNPs, represented by the minor (less frequent) allele. Bars are color-coded according to SNP type: synonymous (green), non-synonymous (orange), nonsense (red) and intergenic (grey). C) The expected and observed number of within-host mutations per gene (solid and hatched bars respectively), combined across participants A–M, Q and R.

We investigated the role of natural selection by comparing synonymous and non-synonymous polymorphism within and between hosts. Our analyses revealed that purifying selection is the dominant selective force acting on *S. aureus* over short timescales. We discovered a total of 37 synonymous SNPs and 94 non-synonymous SNPs within hosts. We estimate that the rate of non-synonymous mutation is 4.6 times higher than that of synonymous mutation in *S. aureus* based on codon usage in MRSA252 and the observed transition:transversion ratio in non-coding SNPs. Allowing for this gives a *d_N_*/*d_S_* of 0.55 within hosts, significantly below 1, indicating the dominance of purifying selection against changes in encoded proteins (binomial test, *p* = 0.004). We discovered a total of 499 synonymous SNPs and 654 non-synonymous SNPs between hosts and their respective clonal complex-specific reference genomes, corresponding to a *d_N_*/*d_S_* of 0.28, also significantly below 1 (binomial test, *p*<0.001). We used the test of McDonald and Kreitman [50] to compare *d_N_*/*d_S_* within and between hosts. We found that *d_N_*/*d_S_* between hosts was significantly lower than within hosts (*p*<0.001), consistent with the action of purifying selection, which is expected to purge deleterious mutations more efficiently over longer timescales (Figure 4A) (see e.g. [51]–[53]).

There was further evidence for the influence of purifying selection in the sample frequencies of synonymous and non-synonymous polymorphisms (Figure 4B), which differed significantly (G test of the folded site frequency spectrum, *p* = 0.028). Among polymorphisms with a minor allele count (MAC) of one (*i.e.* SNPs at which the less frequent allele was observed in only one colony), 48 were non-synonymous and 7 were synonymous, which gave *d_N_*/*d_S_* = 1.49, much higher than the average within hosts, but not significantly different from the *d_N_*/*d_S_* = 1 expected under neutrality (binomial test, *p* = 0.382). There were 46 non-synonymous polymorphisms with MAC >1, compared to 28 synonymous polymorphisms, which gave *d_N_*/*d_S_* = 0.36, significantly different to 1 (binomial test, *p*<0.001). This tendency for non-synonymous polymorphisms to segregate at lower frequencies than synonymous polymorphisms is consistent with purifying selection (G test, *p* = 0.001). The distribution of MAC for non-coding SNPs was intermediate between synonymous and non-synonymous SNPs. In particular, the unusual rarity of singletons (MAC = 1) among synonymous polymorphisms was not repeated in intergenic SNPs, suggesting a degree of purifying selection acting on intergenic SNPs intermediate between that affecting synonymous and non-synonymous SNPs.

Lieberman and colleagues [28] reported strong evidence for parallel adaptation during within-host bacterial evolution among 14 cystic fibrosis sufferers infected with *Burkholderia dolosa*. Using a similar analysis of the number of mutations per gene aggregated over hosts (see Figure 4a in ref. [28]), we did not find a significant difference between the expected and observed number of mutations per gene (by simulation, *p* = 0.413). This was consistent with our other results suggesting that purifying selection is the dominant force shaping within-host evolution of *S. aureus*. To further test this hypothesis we included in our analysis an additional 101 *S. aureus* colonies isolated from two longitudinally sampled asymptomatic nasal carriers, participants Q and R, which we previously reported [29]. Figure 4C shows the observed numbers of genes harboring 0, 1, 2 and 3 or more mutations, aggregated over participants A–M, Q and R, alongside the expected numbers in each category. There were 16 genes with two mutations, higher than the expectation of 9.16, although the statistical significance of this excess was marginal (by simulation, *p* = 0.045). Taking into account the small magnitude of the excess and its marginal significance, we conclude that adaptive evolution during nasal carriage of *S. aureus* must be rare. Nevertheless, we noted that in seven of the 17 total genes with multiple mutations, the multiple hits occurred within the same host. That they occurred on different branches of the within-host tree excludes homologous recombination as a likely explanation for this observation. Table 3 lists all the genes with two or more mutations across individuals, notable among which are two genes encoding surface anchored proteins and an enterotoxin gene.

**Table 3:** Genes affected by multiple mutations among hosts A–M, Q and R.

		SNPs/indels		
CDS in MRSA252	Function	Syn	Non-syn	Stop
SAR0180	Non-ribosomal peptide synthetase	1	1	0
SAR0329	PTS regulator	0	2	0
SAR0457	Hypothetical protein	1	1	0
SAR0466	MutT domain-containing protein	1	1	0
SAR0471	Glutamate synthase	0	2	0
SAR0527	ATP:guanido phosphotransferase	0	2	0
SAR0558	Hypothetical protein	2	0	0
SAR1292	FtsK/SpoIIIE family protein	1	1	0
SAR1447	Very large surface anchored protein	0	2	0
SAR1789	Acetate kinase	1	1	0
SAR1841	Surface anchored protein	0	3	0
SAR1916	Enterotoxin	0	1	1
SAR1965	ThiJ/PfpI family protein	0	1	1
SAR2292	Hyaluronate lyase precursor	1	1	0
SAR2472	Proton/sodium-glutamate symport protein	1	1	0
SAR2691	Betaine aldehyde dehydrogenase	0	1	1
SAR2726	Hypothetical protein	1	1	0

### Fluctuating population dynamics characterize nasal carriage

We sampled genomes from a single time point in each individual, representing a snapshot of the evolutionary process within each host. However, the genomic data showed evidence of temporal fluctuations in population size during carriage. In the frequency spectrum of polymorphisms, synonymous singleton polymorphisms (MAC  = 1) were unusually rare compared with doubletons (MAC  = 2, Figure 4B), and the within-host genealogies exhibited vivid differences in diversity ranging from the complete absence of within-host variation through to highly diverse populations harboring very long branches (Figure 1). Since carriage of *S. aureus* is often intermittent [54], [55], it follows that the population must undergo expansion during colonization, and contraction during clearance. We therefore modeled within-host population dynamics as fluctuating in size using a simple deterministic model that encompassed growth and decline. We assumed the mutation rate was constant and the same across hosts. To improve statistical power, we fitted the model of longitudinal dynamics to all 13 carriage populations, allowing them to differ only in the stage of the cycle of expansion and contraction at the time of sampling. We used the model to estimate the extent of population fluctuations during nasal carriage, the timescale of such fluctuations, and whether the bacterial population was growing or declining in each host at the point of sampling.

**Figure 5:**
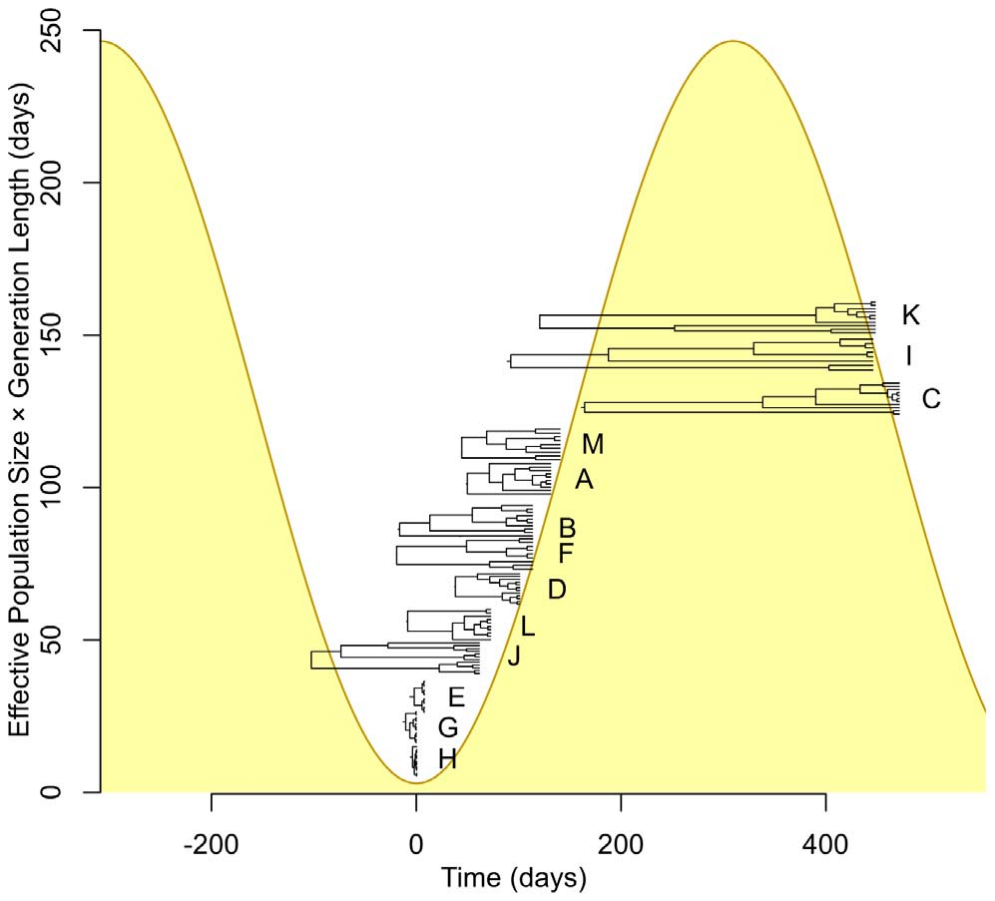
Within-host genealogies inferred under a model of nasal population size fluctuations during carriage. Assuming a model of population growth and decline, the bacterial genealogy within each host A-M was estimated using an extension to BEAST. The maximum clade credibility tree is shown with the tips aligned to the estimated time in the cycle of growth and decline when sampling took place. The shaded area shows the fitted “skyline” for within-host population size (measured as the product of the effective population size and generation length), assuming a mutation rate of 2.7 mutations per megabase per year [29]. Time is arbitrarily measured relative to the trough in population size.

Figure 5 illustrates the cycle of expansion and contraction for the fitted model. The estimated genealogies relating colonies within each host are superimposed on the same time axis, with the tips aligned to the estimated position in the cycle of population size change at which sampling took place. Carriage populations with low and intermediate SNP diversity were estimated to lie in the trough in population size, or shortly afterwards as expansion begins. Populations with high diversity were inferred to have been sampled at some point after the peak in population size, as the population begins to crash. We estimated the period of the fluctuations to be 618 days (95% credible interval 414–976 days). The vertical axis in Figure 5 is *N_e_g*, the product of effective population size *N_e_*, corresponding to the census size of an idealized population [56], and generation time *g*. We estimated *N_e_g* to vary between a minimum of 3.0 days (95% C.I. 0.05–21.8 days) and a maximum of 250 days (95% C.I. 165–410 days). Assuming a doubling time of ca. 90 minutes [57] implies that the effective size of the reproductively viable population of *S. aureus* fluctuates between 50 and 4000 during nasal carriage, *i.e.* across two orders of magnitude.

### Evidence for recent transmission

An important application of bacterial whole-genome sequencing will be to monitor the extent of transmission in hospitals, public institutions and the community. Recent estimates of the molecular clock rate in *S. aureus* provide an entry point for the interpretation of genomic divergence in terms of the time since the most recent common ancestor (MRCA) of two or more isolates [29], [58]. However, knowledge of the transmission rate is necessary to convert this into an estimate of the number of links in the transmission chain. We developed a simple population genetics method, based on coalescent theory of metapopulations [59] (see Methods), to estimate the length of transmission chains separating two bacterial genomes sampled from different hosts. Using estimates of evolutionary and epidemiological parameters from a number of studies including this one [29], [60]–[63], we assessed the evidence for recent transmission between the 13 participants in our carriage study.

The key evolutionary parameters were the molecular clock rate, which we took to be 2.7 mutations per megabase per year [29], and mean within-host diversity, which we found to be 4.12 SNPs (Table 1). The key epidemiological parameters were the prevalence of multiple colonization, which has been estimated at 6.6% for *S. aureus* carriage [60], and the rate of transmission. Assuming prevalence is stable, the transmission rate equals the reciprocal of mean carriage duration. Estimates vary widely from 70 days [61], through 15 months [62] to 40 months [63], with one study detecting stable carriage after eight years [55]. To account for this uncertainty, we explored two scenarios: slow transmission corresponding to mean carriage duration of 40 months and rapid transmission corresponding to mean carriage duration of 10 months.

**Figure 6:**
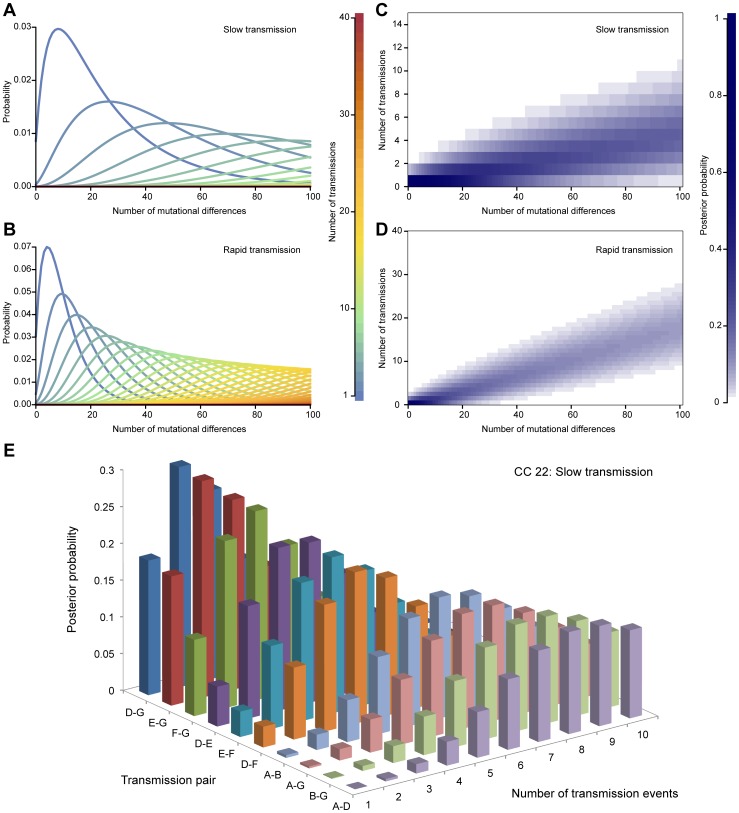
Estimating the number of transmission events from genomic divergence. We used a simple population genetics model to calculate the probability of the number of mutational differences between two bacterial genomes, conditional on the number of transmission events that have occurred since their most recent common ancestor, under A) slow transmission (0.3 transmissions per year) and B) rapid transmission (1.2 transmissions per year). We employed the model to estimate the Bayesian posterior probability of the number of transmission events conditional on the observed number of mutational differences, under C) slow and D) rapid transmission. E) When we applied the model to CC22 genomes under the slow transmission model, we detected evidence for very recent transmission between some pairs of hosts, including the possibility of direct transmission. In A) and B), the lines are color-coded according to the number of transmission events, indicated by the key. In C) and D), the magnitude of the posterior probability is indicated by the intensity of the shading, as shown by the key. In E), the ten pairs of hosts with most evidence for recent transmission are shown. The colors distinguish transmission pairs.

Figure 6A and 6B show, for the slow and rapid transmission scenarios respectively, the probability distributions generated by our model for the number of mutational differences between a pair of genomes conditional on the number of transmission events separating them. Using this model, we computed the posterior distribution of the number of transmission events given the observed number of mutational differences under either scenario, assuming a uniform prior for the length of the transmission chain (Figure 6C and 6D). This shows that the estimated number of transmission events is higher under the rapid transmission scenario, and increases approximately linearly with the number of mutational differences.

We applied the method to estimate the number of transmission events separating the CC22 and CC30 carriers in our study. We adjusted the directly observed number of SNP differences between genomes representing each of our 13 hosts to account for differences in the level of filtering in our SNP calling pipeline, which reduces the observed number of SNP differences, and recombination, which increases it (Table S3). Further details are provided in Text S1. Under the slow transmission scenario, we found evidence for very recent transmission between CC22 carriers. Figure 6E shows the posterior distribution of the number of transmission events separating hosts. We obtained maximum *a posteriori* estimates of just two transmission events separating host pairs D and G (D–G) and E–G (Table S3). However, the 95% credible interval did not exclude direct transmission between hosts D, E, F and G, all of whom were MRSA carriers. Among this group were host pair D–E, the fourth most closely related pair (posterior mode 3 transmissions, 95% credible interval 1–7 transmissions, posterior probability of direct transmission 5.4%), who we later discovered were husband and wife.

## Discussion

In this study we used whole-genome sequencing to investigate the evolutionary dynamics and population genomics of *S. aureus*, a common commensal and major pathogen, during normal asymptomatic carriage. Our study contributes to a body of work that is shedding light on the genomic basis of within-host bacterial evolution [21]–[29], and builds on earlier investigations of within-host variation based on morphological phenotyping and pulsed field gel electrophoresis (e.g. [64]–[66]). We analyzed 131 bacterial colonies sampled from 13 singly colonized hosts in order to better understand the processes by which the raw material for evolution is generated. We reported frequent microvariation in the form of SNPs and short indels from snapshots of within-host diversity. The level of diversity within hosts was uniformly lower than that detected between hosts, supporting our view, initially formed on the basis of evidence from *spa* typing [30], that these nasal carriage populations represented single founding colonization events. The existence of sparse but detectable microvariation is consistent with that previously detected in long-term carriers [22], [29], and we propose that it is a feature typical of staphylococcal nasal carriage. The common existence of within-host microvariation below that detectable by routine methods such as *spa* typing [30] and MLST [31] underlines the utility of whole-genome sequencing for understanding the evolutionary dynamics of bacteria, and in particular genetically monomorphic pathogens [18], [19]. We also observed gross structural variation in the *S. aureus* genome in several hosts, including indels up to 41 kb in length, associated with the activity of mobile elements, similar to patterns of longitudinal evolution observed in cystic fibrosis patients [25], [64], [65]. We saw examples of variation in the presence of bacteriophages homologous to φPVL and φNM3 [44], [47], [48] and the pT48 plasmid [49]. It appears therefore that structural variation, while less common than microvariation, is a relatively frequent occurrence even within singly-colonized nasal carriage populations of *S. aureus*. Investigations in a number of bacterial species suggest that we should expect within-host variation during colonization and infection to be the rule, rather than the exception [20]–[29].

Studies of natural selection during colonization and infection in *Burkholderia dolosa* [28], *Escherichia coli* [23], *Helicobacter pylori* [26] and *Pseudomonas aeruginosa* [21] have reported evidence of adaptive evolution. In contrast, we found scant evidence for adaptation during *S. aureus* nasal carriage, except for a marginal excess of mutations in a small number of genes including those encoding surface proteins and enterotoxin. Rather, we report a fitness landscape dominated by purifying selection, as witnessed by the *d_N_*/*d_S_* ratio. We observed the highest *d_N_*/*d_S_* ratio of 1.49 for within-host singleton SNPs (SNPs with a minor allele sample frequency of one), falling to 0.36 for within-host non-singleton SNPs, and falling further to 0.28 for between-host SNPs. This tendency for the *d_N_*/*d_S_* ratio to decrease as the frequency of a SNP (and hence its expected age [67]) increases is ascribed to the delayed action of purifying selection which tolerates weakly deleterious non-synonymous mutations at low frequencies, but disfavors their drift to high frequencies (see, e.g. [51]–[53]). Similar patterns of time dependency in the *d_N_*/*d_S_* ratio have been reported in various species [51], [52] including *Helicobacter pylori* [24] and, over longer time frames, *Clostridium difficile* and *S. aureus* [14], [68].

Although our observations were based on snapshots of the evolutionary process in individual carriers, we found evidence for fluctuating population dynamics within hosts. Hosts differed in the number and frequency distribution of mutations. Since carriage of *S. aureus* is frequently intermittent [54], [55], we modeled these demographic changes using a model that captures the dynamics of colonization, establishment and eventual clearance. Our analysis suggested the effective size of the *S. aureus* population fluctuates one hundred-fold over the course of colonization, with a periodicity of 20 months. We assumed a constant molecular clock rate among the 13 hosts of 2.7 mutations per megabase per year. This figure, inferred from longitudinal evolution of *S. aureus* within participant P, a nasal carrier who developed invasive disease [29], is within the 95% credible interval inferred from another nasal carrier, participant R (1.08–3.06) [29], and the 95% confidence interval inferred from ST239 isolates over a longer time frame (2.5–4.0) [58]. Further studies are needed to investigate the extent of variation in the molecular clock rate within and between individuals. If there was appreciable variation in the clock rate, we may have underestimated the uncertainty in the demographic parameters, although we would still expect a correlation between within-host diversity and both the date of the within-host MRCA and the within-host effective population size.

Whole-genome sequencing offers unprecedented resolution for reconstructing short-term bacterial evolution, not just within individual hosts, but also along host-to-host transmission chains. Monitoring transmission will be an important application for pathogen whole-genome sequencing [69], [70]. We developed a metapopulation model to assess the evidence for recent transmission among hosts, correcting for recombination using a method based on ClonalFrame [40]. Just as knowledge of the molecular clock rate is vital if SNP differences are to be interpreted in terms of temporal divergence of bacterial lineages, the transmission rate is essential for estimating the number of links in a transmission chain. Current estimates of carriage duration (and hence transmission rates) in *S. aureus* vary widely and suffer from censoring, such that longer studies have tended to produce longer estimates of carriage duration [55], [61]–[63]. Consequently, we explored two rates representative of rapid and slow transmission. Under the slow transmission scenario we detected evidence for very recent transmission between hosts carrying populations of clonal complex 22 MRSA. Although our model ignored a number of complexities including variation in within-host diversity, we think this result is plausible for several reasons. First, the deliberate enrichment of our sample for MRSA carriers (5/13) relative to the population prevalence of 2.5% (9/360) would tend to cause sampling of bacteria more closely related than average. Second, the adjusted SNP differences of 46–92 among hosts D, E, F and G correspond to divergence times of circa 3–6 years, consistent with some of the longer estimates of carriage duration [55], [63]. Third, epidemiological investigation revealed hosts D and E to be a married couple, although no epidemiological links were found between any of the other host pairs for whom direct transmission could not be ruled out. The current study provides a starting point for analysis of recent transmission in bacterial pathogens using whole-genome data. By providing insight into the population dynamics and evolution of *S. aureus* during normal, asymptomatic carriage, we hope it will also serve as a yardstick for future comparisons of within-host evolution in other settings including, importantly, during pathogenesis.

## Methods

### Ethics Statement

Ethical approval for the *S. aureus* carriage study was obtained from the Oxfordshire B Oxfordshire Research Ethics Committee (reference number 08/H0605/102). All participants provided written informed consent.

### Isolate collection and preparation

We surveyed nasal carriage in 1,123 adults attending general medical practices in Oxfordshire, UK. Nasal swabs obtained from study participants were incubated overnight at 37°C in enrichment broth containing 5% NaCl (E and O Laboratories, Bonnybridge, UK), and a 5 mm loopful of broth was plated onto SASelect® chromogenic agar (Bio-Rad, Limerick, Ireland). Colonies were verified as *S. aureus* based on morphology and DNAse, catalase and Staphaurex tests (Standards Unit, Centre for Clinical Infections, 2007), and classified as methicillin susceptible or resistant based on growth surrounding a 1 μg oxacillin antimicrobial susceptibility disc (Becton Dickinson, Oxford, UK). Samples were stored in such a way as to preserve existing genetic diversity: material from ∼50 randomly-selected colonies was pooled and frozen in glycerol at –80°C. Staphylococcal protein A (*spa*) type was determined by Sanger sequencing of the variable X region of the 3′ end of the *spa* gene, using commercially designed primers (spaF 5′-AGACGATCCTTCGGTGAGC-3′ spaR 5′- GCTTTTGCAATGTCATTTACTG-3′). The software Ridom StaphType [30] was used for *spa* sequence analysis. We selected 13 confirmed carriers of the hospital-associated CC22 and CC30 groups including five MRSA carriers in which to study within-host genetic diversity. For each, we isolated 12 colonies from the glycerol stocks following 24 hours growth, emulsified them in 5% NaCl broth, and incubated overnight. DNA was extracted using the Qiagen DNEasy tissue kit (Qiagen, Crawley, UK) according to the manufacturer's directions.

### Genome sequencing and assembly

Multiplex paired-end libraries using 12 indices were prepared from the DNA with a median fragment size of 200 base pairs (bp). Samples were sequenced at the Wellcome Trust Centre for Human Genetics, Oxford on the Illumina GAIIx platform with 51 base paired-end reads, obtaining a median of 94.3-fold genomic coverage (minimum 28.1, maximum 223.1). In 131 of a total of 156 colonies we successfully performed DNA extraction, library preparation and sequencing to a stringent quality standard.

Reference genomes for mapping were obtained from GenBank for CC30 (MRSA252, accession number BX571856, [39]) and from the Wellcome Trust Sanger Institute for CC22 (EMRSA15, ftp://ftp.sanger.ac.uk/pub/pathogens/sa/EMRSA15.dna), and generated *de novo* from one sample per host as follows. For every colony, the genome was assembled using Velvet [71] v1.0.11, with hash (kmer) size and coverage parameters optimized to give the highest number of bases in contigs with length greater than 1 kb. Contigs (75 – 431 per sample, mean 173) were aligned against the MRSA252 sequence using blastn, and concatenated after removal of overlapping ends. The colony with the longest assembled genome in each host was chosen as the host-specific reference sequence. These host-specific references were used for variant calling within each cluster of colonies from an individual host, and annotated using the xBASE bacterial genome annotation service [72].

### Variant calling

For the detection of single nucleotide variants relative to the reference, we used a combination of a reference-based mapping approach via Stampy [73] and a population-based *de novo* assembly approach via Cortex [74], as previously described [75], with additional manual curation for the confirmation of all variants. Reads were mapped to the appropriate reference sequence using Stampy [73] v1.0.11 with no BWA pre-mapping and an expected substitution rate of 0.01. On average 97.1% of reads mapped to the host-specific reference sequence. Non-unique regions were identified by a self-self blast analysis of the reference sequence to locate regions of high internal homology, using megablast parameters (word size 28) to exclude spurious short matches (BLAST+ v2.2.24 [43]). This masked 3.4% of the genome on average. Variants were called using the SAMtools v0.1.12 [76] mpileup command with options -M0 -Q30 -q30 -o40 -e20 -h100 -m2 -D -S. Variants were filtered using the following criteria: (1) the depth of high-quality coverage within the (2.5%, 97.5%) quantiles of the distribution across all sites but with an absolute minimum of five reads at the variant site and at least one read in either direction, (2) no other variant within 12 bp, (3) at least 75% of reads at the site supporting the call, (4) a homozygous call under a diploid model, (5) did not fall in a non-unique region. The false positive rate (where we detect a spurious variant) for our bioinformatics pipeline was previously estimated to be 2.5×10^−9^ per nucleotide [75]. Consequently, we expected less than one false SNP in our study. A mean of 92.4% of the host-specific reference sequence was called in each sample, implying a false negative rate (where we fail to detect a true variant) of 7.6% for singleton SNPs. For SNPs with higher sample frequency, this fraction will be smaller. The bioinformatics pipeline was intentionally designed to have a low false positive rate, at the expense of a somewhat higher false negative rate. The average values for the 2.5% threshold were 46.5 and 97.7 reads when mapping against the clonal complex-specific and host-specific reference genomes respectively. In only one sample out of 131 did the threshold fall as low as five reads for either reference. We identified SNPs by comparing calls across isolates from the same host. Each called SNP was verified by visual inspection of aligned reads for at least one instance of each non-reference allele. Where calls at putative SNPs had been filtered out in some samples, visual inspection was used to validate the SNP and, where possible, a manual call was made. Figure S1 shows, for every singleton SNP, the number of reads supporting each base call in every colony from the host in question.

We used Cortex [74] to identify single nucleotide variants and short indels from a joint *de novo* assembly of each cluster of samples from an individual host. The results were filtered to remove repetitive regions. 154 SNPs were identified by mapping and Cortex, three by mapping only and five by Cortex only. To identify large deletions relative to the Velvet-assembled host-specific reference sequences, the genomes mapped using Stampy were scanned for regions of at least 1 kb where 500 bp or more were covered by no reads. Reads that could not be mapped to the host-specific reference were assembled using Velvet with a constant hash size of 31. Contigs greater than 1 kb in length were considered to be insertions relative to the reference. Large indels were confirmed by aligning the Velvet assembly of one of the genomes containing the putative indel to the host-specific reference sequence using progressiveMauve [41], after ordering the contigs of both genomes using the Mauve Contig Mover [42].

### Genealogical relationships

We used a permutation test [32], implemented as part of omegaMap [36], to detect evidence of recombination within hosts that is based on the correlation between physical distance and linkage disequilibrium, as measured by the *r* ^2^ statistic [37]. We detected homoplasies using the four gamete test [38]. Since we found no evidence for homoplasy or recombination within hosts, we inferred the tree topology and branch lengths of the genealogies relating colonies sampled within the same host using maximum likelihood (ML) under the assumption of no repeat mutation and homogeneous mutation rates. We inferred genealogical relationships between hosts using ClonalFrame [40], which accounts for possible repeat mutation and recombination. We analyzed one sequence per host (the host-specific reference genome) based on mapping to the clonal complex-specific reference (EMRSA15 for CC22 and MRSA252 for CC30).

### Natural selection

To detect differences in the strength and mode of selection within versus between hosts, we employed the McDonald-Kreitman test [50], tabulating the number of synonymous and non-synonymous SNPs within each host A–M, and the total number of synonymous and non-synonymous point mutations occurring between host-specific and clonal complex-specific reference sequences. To compare the effects of selection acting on different types of polymorphism, we cross-tabulated the minor allele count of each SNP (defined as the sample frequency of the less abundant allele) by SNP type (synonymous, non-synonymous, premature stop and non-coding). We tested for differences in this distribution (known as the folded site frequency spectrum) between SNP types using the G test [77].

To detect evidence for excess diversity among some genes, we tabulated the number of genes with 0, 1, 2 and 3 or more mutations, aggregated across the 13 individuals newly sequenced here (A–M) and two previously reported individuals from the same carriage study (Q and R, [29]). We calculated the expected counts conditional on the total number of mutations in coding sequences, corrected for the distribution of coding sequence lengths in the MRSA252 reference genome (on which our xBASE annotations were based). To test for a significant deviation from the expected distribution, we calculated a test statistic 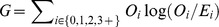, where *O_i_* and *E_i_* were the observed and expected number of genes with *i* mutations, and simulated a distribution for *G* under the null hypothesis that mutations are independent and occur with probability proportional to gene length, computing a one-tailed *p*-value for *G* greater than or equal to that observed.

### Population size fluctuations

Population dynamics of growth and decline within individual hosts were modeled using a harmonic function for the effective population size over time that was based on the sine squared function, which is constrained to be positive:
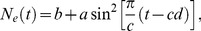
(1)


where *a* is the amplitude (or range) of the oscillations, *b* is the baseline (or minimum) population size, *c* is the period of the oscillations and *d* is the point in the cycle that sampling took place (0 ≤ *d* ≤ 1, where the population is expanding at sampling if 0<*d*<0.5). The demographic model was implemented in Java as an extension to BEAST [78], which requires calculation of the cumulative inverse population size [79], defined for this model as:
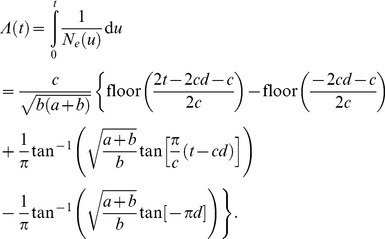
(2)


To fit the model to hosts A–M, we assumed that parameters *a*, *b* and *c* are common to all hosts, who differ only by *d* (the point of the cycle at which sampling took place). Exponential priors with mean 100 were assumed for parameters *a*, *b* and *c*, with time measured in days, and uniform priors on *d*. We employed the HKY mutation model [80] assuming a mutation rate of 2.72 per megabase per year [29], [58], a uniform prior on nucleotide frequencies, and a log-normal prior on *κ* (transition:transversion ratio) with mean 1 and standard deviation 1.25 on the logarithmic scale. To help infer the ancestral allele at each SNP, we constructed an outgroup sequence for each host from the homologous positions in the clonal complex-specific reference genome. An improper uniform prior was used for the coalescence time with the outgroup. All SNPs and 1% of the fixed differences between each host-specific and clonal complex-specific reference genome were included in the analysis, with a 100-fold linear correction made to the mutation rate. Two runs of the Markov chain Monte Carlo algorithm were performed of 10 million iterations each, sampled every 1,000 iterations with a burn-in of 100,000 iterations removed before merging the chains to obtain final results.

### Length of transmission chains

We developed a population genetics model, based on the theory of metapopulations [59], to obtain a likelihood function for the number of transmission events given the observed number of SNP differences between a pair of contemporaneously sampled genomes. We assumed a susceptible-infectious-susceptible (SIS) model, where the proportion of hosts infected with a single or multiple strains at time *t* are 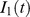 and 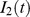 respectively. The total rate of new single infections is 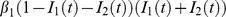 and the total rate of new multiple infections is 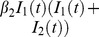, where *β*_1_ and *β*_2_ are transmission coefficients. The total rates at which singly and multiply infected hosts clear infection and return to the susceptible class are 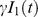 and 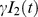 respectively. In our metapopulation analogy, hosts are interpreted as demes that are either colonized (infected) or not. The rate of colonization (new single infection) per infected host is *E*_0_ and the rate of migration (new multiple infection) per infected host is *M* [59]. We assume that the number of infected hosts is large and the number of founding genotypes for each new infection event is one. Assuming further that the number of infections is at dynamic equilibrium, we find that 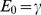 and 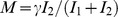, where *I*_1_ and *I*_2_ are the equilibrium proportion of singly and multiply infected hosts. We differ from Wakeley's [59] parameterization in that we measure the rates *E*_0_ and *M* in conventional time units (*e.g.* per year), rather than in coalescent time units. The conversion factor is the average within-host coalescence rate, *λ*.

The time, *T*, to the common ancestor of a pair of genomes sequenced from two hosts separated by *X* transmission events (where *X* = 0 is interpreted as sampling from the same host) is the sum of the time *V* during which the two ancestral lineages were in different hosts and the time *U* during which the two ancestral lineages were in the same host but had not yet coalesced. Under the metapopulation model, *U* follows an exponential distribution with rate 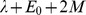. Assuming the number of infected hosts is large and *X* is small, *V* follows a gamma distribution with shape parameter *X* and rate 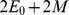. If mutation is rare and occurs at rate *μ* per genome then the number of mutations, *W* and *Z*, that accumulate during time intervals *U* and *V* follow negative binomial distributions with parameters 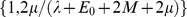 and 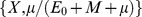 respectively. The probability of observing *Y* mutational differences between two genomes separated by *X* transmission events (*i.e.* the likelihood of *X* given *Y*) is therefore
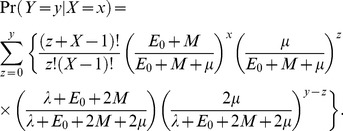
(3)


The parameter values can be populated using knowledge of the average duration of infection, the relative frequency of multiple infections, the genomic mutation rate and the average diversity of singly infected hosts, the latter via the equation 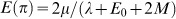. We adjusted the observed number of SNP differences between the host-specific reference genomes of each pair of hosts to correct for (i) the proportion of sites filtered out by our variant calling pipeline and (ii) the effects of recombination. Further details are provided in Text S1.

### Data access

Databases: Data from this study will be made available on publication from the European Nucleotide Archive Sequence Read Archive at http://www.ebi.ac.uk/ena/data/view/ERP001219.

## Supporting Information

Figure S1 **The number of reads supporting base calls for singleton SNPs.** For each singleton SNP, the number of reads supporting each base call (A: red, C: blue, G: green, T: yellow) in the forward (solid) or reverse (hashed) direction is shown for each colony from the host in question. Participant ID and position in the concatenated host-specific reference genome are indicated above each panel. The vertical axis (number of reads supporting the call) is the same for all panels, allowing variation in average depth of coverage to be seen.
(PDF)

Table S1 **Details of variants discovered.**
(DOC)

Table S2 **Evidence for recombination within and between hosts.**
(DOC)

Table S3 **Evidence of transmission between hosts.**
(DOC)

Text S1 **Adjusting Observed SNP Differences for Call Rates and Recombination.**
(DOC)
