# Supplementary figures and images for: Within-Host Evolution of Staphylococcus aureus during Asymptomatic Carriage
Source: PLoS One. 2013 May 1;8(5):e61319. doi: 10.1371/journal.pone.0061319 (PMC3641031; doi:10.1371/journal.pone.0061319)

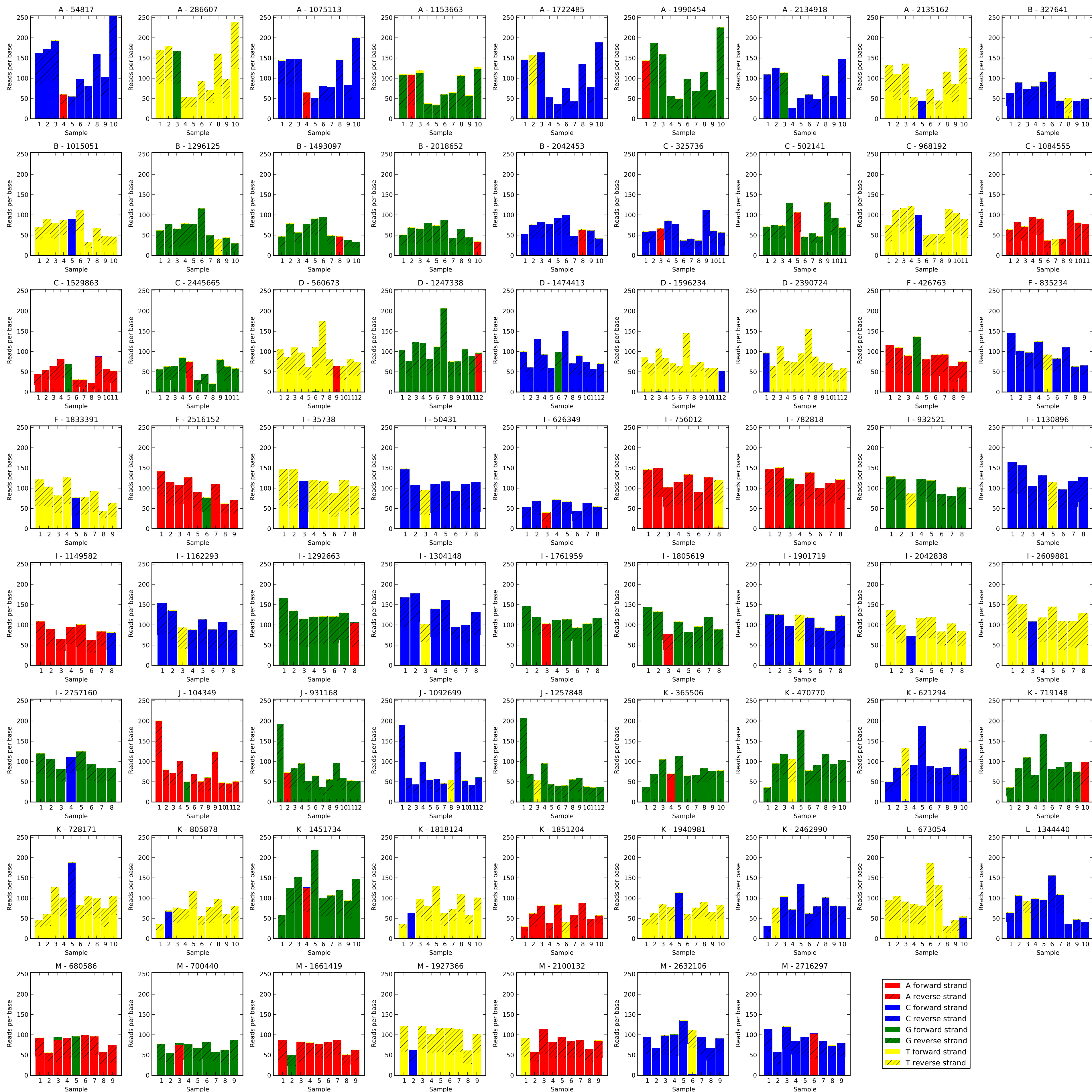

Supplement: Figure S1 — The number of reads supporting base calls for singleton SNPs. For each singleton SNP, the number of reads supporting each base call (A: red, C: blue, G: green, T: yellow) in the forward (solid) or reverse (hashed) direction is shown for each colony from the host in question. Participant ID and position in the concatenated host-specific reference genome are indicated above each panel. The vertical axis (number of reads supporting the call) is the same for all panels, allowing variation in average depth of coverage to be seen. (PDF) [file pone.0061319.s001.pdf]
