# Supplementary material for: Within-Host Evolution of Staphylococcus aureus during Asymptomatic Carriage
Source: PLoS One. 2013 May 1;8(5):e61319. doi: 10.1371/journal.pone.0061319 (PMC3641031; doi:10.1371/journal.pone.0061319)
Supplement: Table S1 — Details of variants discovered. (DOC) [file pone.0061319.s002.doc]

Table S1. Details of variants discovered

| **Parti-cipant** | **Position in host-specific reference** | **Ref Base** | **Non-ref Base** | **Type** | **Effect** | **CDS in MRSA252** | **Gene** | **Ref codon** | **Non-ref codon** | **CDS function** | **COG Group** |
| --- | --- | --- | --- | --- | --- | --- | --- | --- | --- | --- | --- |
| A | 54817 | C | A | SNP | Non-synonymous | SAR0351 | *thl* | GTA | TTA | acetyl-CoA acetyltransferase | I |
| A | 286607 | T | G | SNP | Non-synonymous | SAR2559 |  | ATT | CTT | short chain dehydrogenase | IQR |
| A | 1075113 | C | A | SNP | Non-synonymous | SAR0935 |  | GGA | GTA | hypothetical protein | R |
| A | 1153663 | G | A | SNP | Non-coding |  |  |  |  |  |  |
| A | 1722485 | C | T | SNP | Non-synonymous | SAR1203 | *recG* | ACG | ATG | ATP-dependent DNA helicase RecG | KL |
| A | 1990454 | G | A | SNP | Non-synonymous | SAR0515 | *folP* | CCT | CTT | dihydropteroate synthase | H |
| A | 2134918 | C | G | SNP | Non-synonymous | SAR1789 | *ackA* | GCG | GGG | acetate kinase | C |
| A | 2135162 | T | C | SNP | Synonymous | SAR1789 | *ackA* | CCT | CCC | acetate kinase | C |
| A | 871371 | G | GG | Indel | Non-coding |  |  |  |  |  |  |
| B | 327641 | C | T | SNP | Non-synonymous | SAR0340 |  | GCA | ACA | putative lipoprotein | P |
| B | 515817 | T | C | SNP | Synonymous | SAR2580 | *fnbA* | GAT | GAC | fibronectin-binding protein precursor | - |
| B | 702686 | G | A | SNP | Non-coding |  |  |  |  |  |  |
| B | 1015051 | T | C | SNP | Non-synonymous | SAR2265 |  | ACG | GCG | hypothetical protein | S |
| B | 1212082 | G | A | SNP | Synonymous | SAR2472 | *gltT* | GAC | GAT | putative proton/sodium-glutamate symport protein | C |
| B | 1296125 | G | T | SNP | Non-synonymous | SAR1965 |  | GAC | TAC | ThiJ/PfpI family protein | R |
| B | 1493097 | G | A | SNP | Non-synonymous | SAR2126 | *agrA* | CGC | TGC | autoinducer sensor protein response regulator protein | KT |
| B | 1861142 | C | T | SNP | Non-synonymous | SAR1592 |  | GCA | GTA | hypothetical protein | EH |
| B | 2018652 | G | A | SNP | Non-synonymous | SAR0559 |  | GAA | AAA | branched-chain amino acid aminotransferase | EH |
| B | 2042453 | C | A | SNP | Non-synonymous | SAR2628 | *clpL* | CAA | AAA | putative ATP-dependent protease ATP-binding subunit ClpL | O |
| B | 2645208 | A | G | SNP | Non-synonymous | SAR0969 |  | AAT | GAT | hypothetical protein | - |
| C | 16330 | G | A | SNP | Non-synonymous | SAR2453 |  | CTT | TTT | hypothetical protein | V |
| C | 59948 | G | A | SNP | Non-coding |  |  |  |  |  |  |
| C | 146698 | G | A | SNP | Non-synonymous | SAR1293 |  | GAA | AAA | hypothetical protein | U |
| C | 325736 | C | A | SNP | Non-coding |  |  |  |  |  |  |
| C | 332256 | G | A | SNP | Non-coding |  |  |  |  |  |  |
| C | 463314 | G | A | SNP | Synonymous | SAR0144 |  | GAC | GAT | putative ABC transport ATP-binding protein | P |
| C | 502141 | G | A | SNP | Non-synonymous | SAR0813 | *uvrA* | ACA | ATA | excinuclease ABC subunit A | L |
| C | 506376 | A | G | SNP | Non-coding |  |  |  |  |  |  |
| C | 789093 | T | C | SNP | Non-synonymous | SAR2281 | *lacE* | GTC | GCC | PTS system, lactose-specific IIBC component | G |
| C | 801185 | G | T | SNP | Non-synonymous | SAR2272 |  | GCT | GAT | hypothetical protein | Q |
| C | 913513 | T | C | SNP | Non-synonymous | SAR0241 |  | ATA | ATG | putative PTS transport system, IIB component | G |
| C | 968192 | T | C | SNP | Non-synonymous | SAR0198 |  | ATT | ACT | ABC transporter ATP-binding protein | R |
| C | 1014755 | G | T | SNP | Synonymous | SAR0166 | *capP* | ACC | ACA | capsular polysaccharide synthesis enzyme | M |
| C | 1084555 | A | T | SNP | Non-synonymous | SAR0424 |  | GAG | GTG | superantigen-like protein | - |
| C | 1112036 | G | A | SNP | Non-synonymous | SAR0457 |  | GCG | ACG | hypothetical protein | R |
| C | 1118998 | A | G | SNP | Non-synonymous | SAR0463 |  | GAG | GGG | putative lipoprotein | P |
| C | 1124917 | C | T | SNP | Synonymous | SAR0470 |  | AGG | AGA | LysR family regulatory protein | K |
| C | 1212896 | T | C | SNP | Synonymous | SAR1143 |  | ACA | ACG | carbamate kinase | E |
| C | 1529863 | A | G | SNP | Synonymous | SAR1377 |  | GGT | GGC | ImpB/MucB/SamB family protein | L |
| C | 1769901 | G | A | SNP | Synonymous | SAR0230 |  | GCG | GCA | putative extracellular solute-binding lipoprotein | E |
| C | 1867813 | C | T | SNP | Non-synonymous | SAR0326 | *ulaA* | GCA | ACA | ascorbate-specific PTS system enzyme IIC | S |
| C | 2070020 | G | A | SNP | Non-synonymous | SAR1425 | *sucA* | CCA | TCA | 2-oxoglutarate dehydrogenase E1 component | C |
| C | 2445665 | G | A | SNP | Non-synonymous | SAR1839 |  | CCA | CTA | putative polysaccharide biosynthesis protein | R |
| C | 2513965 | G | A | SNP | Synonymous | SAR1193 |  | TTG | TTA | hypothetical protein | J |
| C | 67058 | T | TTAAATTTAT | Indel | Non-coding |  |  |  |  |  |  |
| C | 249912 | A | AA | Indel | Non-coding |  |  |  |  |  |  |
| C | 1478082 | TT | T | Indel | Non-coding |  |  |  |  |  |  |
| C | 1784611 | AATTCATGTTATA | A | Indel | Non-coding |  |  |  |  |  |  |
| C | 2041595 | T | TT | Indel | Non-coding |  |  |  |  |  |  |
| C | 2265870 | TT | T | Indel | Frameshift  No premature stop | SAR1660 |  |  |  | coproporphyrinogen III oxidase | H |
| D | 560673 | T | A | SNP | Non-synonymous | SAR2287 | *lacR* | CAT | CTT | lactose phosphotransferase system repressor | KG |
| D | 1247338 | G | A | SNP | Non-synonymous | SAR2727 |  | CCT | TCT | hypothetical protein | M |
| D | 1474413 | C | G | SNP | Non-synonymous |  |  | ACT | AGT |  | - |
| D | 1596234 | T | C | SNP | Non-synonymous | SAR1292 |  | ACA | GCA | FtsK/SpoIIIE family protein | D |
| D | 2377740 | G | A | SNP | Non-synonymous | SAR1218 |  | GGT | AGT | hypothetical protein | S |
| D | 2390724 | T | C | SNP | Synonymous | SAR1208 | *acpP* | TTA | TTG | acyl carrier protein | IQ |
| F | 154858 | C | T | SNP | Synonymous |  |  | GGC | GGT |  | - |
| F | 302474 | C | T | SNP | Synonymous | SAR0277 |  | AAG | AAA | hypothetical protein | - |
| F | 368269 | T | C | SNP | Non-synonymous | SAR0343 |  | TAG | TGG | putative Sec-independent protein translocase protein | U |
| F | 426763 | A | G | SNP | Non-coding |  |  |  |  |  |  |
| F | 474954 | C | A | SNP | Non-synonymous | SAR0471 | *gltA* | GCA | GAA | glutamate synthase, large subunit | E |
| F | 835234 | C | T | SNP | Non-synonymous | SAR0859 |  | AGT | AAT | OsmC-like protein | O |
| F | 1833391 | T | C | SNP | Non-synonymous | SAR1835 | *dat* | GAA | GGA | D-alanine aminotransferase | EH |
| F | 2117653 | C | T | SNP | Synonymous | SAR2143 | *ilvC* | GAC | GAT | ketol-acid reductoisomerase | EH |
| F | 2243291 | A | G | SNP | Synonymous | SAR2264 |  | GAA | GAG | hypothetical protein | - |
| F | 2516152 | A | G | SNP | Non-coding |  |  |  |  |  |  |
| F | 489647 | TT | T | Indel | Non-coding |  |  |  |  |  |  |
| F | 1899637 | TT | T | Indel | Frameshift  Premature stop | SAR1916 |  |  |  | enterotoxin | - |
| F | 1950785 | CC | C | Indel | Non-coding |  |  |  |  |  |  |
| I | 25642 | C | A | SNP | Non-coding |  |  |  |  |  |  |
| I | 35738 | T | C | SNP | Non-synonymous | SAR2454 | *mqo1* | GAA | GGA | malate:quinone oxidoreductase | R |
| I | 50431 | C | T | SNP | Non-synonymous | SAR2466 | *scrA* | GCA | ACA | PTS system, sucrose-specific IIBC component | G |
| I | 166075 | T | C | SNP | Non-synonymous | SAR0108 |  | ACC | GCC | putative peptidase | R |
| I | 175090 | T | C | SNP | Synonymous | SAR0103 |  | CCA | CCG | hypothetical protein | L |
| I | 248378 | T | C | SNP | Synonymous | SAR2723 |  | GTA | GTG | N-acetylmuramoyl-L-alanine amidase | NU |
| I | 350968 | C | A | SNP | Non-coding |  |  |  |  |  |  |
| I | 492786 | C | A | SNP | Non-coding |  |  |  |  |  |  |
| I | 553345 | C | A | SNP | Non-synonymous | SAR1070 | *pdhD* | GCT | TCT | dihydrolipoamide dehydrogenase | C |
| I | 626349 | C | A | SNP | Non-coding |  |  |  |  |  |  |
| I | 756012 | A | T | SNP | Non-synonymous | SAR0494 | *ksgA* | AAA | ATA | dimethyladenosine transferase | J |
| I | 782818 | A | G | SNP | Non-synonymous | SAR0808 | *prfB* | TAA | CAA | peptide chain release factor 2 | J |
| I | 867652 | C | T | SNP | Non-synonymous | SAR2184 |  | GCT | ACT | hypothetical protein | - |
| I | 882274 | T | C | SNP | Non-synonymous | SAR2201 | *glyA* | AAT | AGT | serine hydroxymethyltransferase | E |
| I | 932521 | G | T | SNP | Non-synonymous | SAR1986 |  | GCT | GAT | ImpB/MucB/SamB family protein | L |
| I | 1130896 | C | T | SNP | Non-synonymous | SAR0201 | *rlp* | GTT | ATT | RGD-containing lipoprotein | E |
| I | 1149582 | A | C | SNP | Non-coding |  |  |  |  |  |  |
| I | 1162293 | C | T | SNP | Non-synonymous | SAR0180 |  | GAA | AAA | putative non-ribosomal peptide synthetase | Q |
| I | 1163077 | C | A | SNP | Synonymous | SAR0180 |  | ACG | ACT | putative non-ribosomal peptide synthetase | Q |
| I | 1268577 | G | A | SNP | Non-synonymous | SAR1251 |  | GCA | GTA | hypothetical protein | R |
| I | 1292663 | G | A | SNP | Synonymous | SAR1231 | *codY* | GGC | GGT | transcriptional repressor CodY | K |
| I | 1304148 | C | T | SNP | Non-synonymous | SAR1222 |  | CGT | CAT | succinyl-CoA synthetase subunit alpha | C |
| I | 1544545 | T | C | SNP | Non-coding |  |  |  |  |  |  |
| I | 1597592 | G | A | SNP | Non-synonymous | SAR1493 |  | ATG | ATA | hypothetical protein | S |
| I | 1720709 | T | C | SNP | Synonymous | SAR1336 |  | GGT | GGC | hypothetical protein | - |
| I | 1761959 | G | A | SNP | Non-synonymous | SAR1450 | *tdcB* | GCA | GTA | threonine dehydratase | E |
| I | 1803543 | A | C | SNP | Synonymous | SAR0743 |  | ATA | ATC | putative sodium:sulfate symporter protein | P |
| I | 1805619 | G | A | SNP | Premature stop | SAR0744 |  | TGG | TAG | putative DNA photolyase | L |
| I | 1892962 | T | C | SNP | Non-synonymous | SAR1567 | *srrB* | ATT | GTT | sensor kinase protein | T |
| I | 1901719 | C | T | SNP | Non-synonymous | SAR1579 |  | GCA | GTA | putative pyrroline-5-carboxylate reductase | E |
| I | 1925969 | G | A | SNP | Synonymous | SAR1601 | *xseA* | GAC | GAT | exodeoxyribonuclease VII large subunit | L |
| I | 2042838 | T | C | SNP | Non-coding |  |  |  |  |  |  |
| I | 2407903 | C | T | SNP | Non-synonymous | SAR2489 | *nasD* | ACA | ATA | nitrite reductase large subunit | C |
| I | 2424868 | C | T | SNP | Non-synonymous | SAR2472 | *gltT* | CCA | TCA | putative proton/sodium-glutamate symport protein | C |
| I | 2468350 | G | A | SNP | Non-synonymous | SAR0429 | *set5* | CGT | CAT | superantigen-like protein | - |
| I | 2469440 | A | G | SNP | Non-synonymous | SAR0431 | *set4* | ATG | GTG | superantigen-like protein | - |
| I | 2517416 | G | A | SNP | Non-coding |  |  |  |  |  |  |
| I | 2609881 | T | C | SNP | Non-synonymous | SAR0329 |  | CAC | CGC | PTS regulator | K |
| I | 2757160 | G | C | SNP | Non-coding |  |  |  |  |  |  |
| I | 2781677 | A | G | SNP | Non-coding |  |  |  |  |  |  |
| I | 713360 | AA | A | Indel | Frameshift  Premature stop |  |  |  |  |  |  |
| I | 823222 | CC | C | Indel | Non-coding |  |  |  |  |  |  |
| I | 1168449 | AA | A | Indel | Non-coding |  |  |  |  |  |  |
| J | 39230 | T | C | SNP | Non-coding |  |  |  |  |  |  |
| J | 104349 | A | G | SNP | Non-synonymous | SAR0196 |  | GAA | GGA | putative type I restriction enzyme | V |
| J | 556181 | C | T | SNP | Non-synonymous | SAR1252 |  | CGT | CAT | putative DNA translocase (FtsK/SpoIIIE family protein) | D |
| J | 719468 | A | G | SNP | Synonymous | SAR1028 |  | ATT | ATC | hypothetical protein | - |
| J | 826209 | T | C | SNP | Non-coding |  |  |  |  |  |  |
| J | 931168 | G | A | SNP | Non-synonymous | SAR0354 |  | GCT | ACT | bifunctional homocysteine S-methyltransferase/5,10-methylenetetrahydrofolate reductase protein | E |
| J | 958077 | C | A | SNP | Non-synonymous | SAR0329 |  | CCA | CAA | PTS regulator | K |
| J | 1092699 | C | T | SNP | Synonymous | SAR2177 |  | TTC | TTT | putative cardiolipin synthetase | I |
| J | 1257848 | G | T | SNP | Non-coding |  |  |  |  |  |  |
| J | 1549136 | T | C | SNP | Non-synonymous | SAR0861 |  | TTG | TCG | nitroreductase family protein | C |
| J | 1639600 | C | T | SNP | Non-synonymous | SAR0958 | *appF* | GGT | GAT | putative oligopeptide transport ATP-binding protein | E |
| J | 1742192 | T | C | SNP | Non-synonymous | SAR2795 |  | ATA | ACA | putative DNA-binding protein | K |
| J | 1841285 | A | G | SNP | Non-synonymous | SAR2544 |  | TAC | CAC | ABC transporter ATP-binding protein | R |
| J | 2208436 | C | T | SNP | Synonymous | SAR0558 |  | GAC | GAT | hypothetical protein | MG |
| J | 2491184 | A | G | SNP | Non-synonymous | SAR0311 |  | GTT | GCT | sodium:solute symporter family protein | ER |
| J | 1481748 | T | TT | Indel | Non-coding |  |  |  |  |  |  |
| J | 2141022 | ATACATATTATAATTTAA | A | Indel | Non-coding |  |  |  |  |  |  |
| J | 2210776 | GG | G | Indel | Frameshift  No premature stop |  |  |  |  |  |  |
| K | 79377 | G | A | SNP | Non-synonymous | SAR0184 | *argJ* | GCA | ACA | bifunctional ornithine acetyltransferase/N-acetylglutamate synthase protein | E |
| K | 227815 | G | A | SNP | Non-coding |  |  |  |  |  |  |
| K | 231253 | G | A | SNP | Non-synonymous | SAR2691 | *cudA* | CGC | TGC | putative betaine aldehyde dehydrogenase | C |
| K | 313335 | T | C | SNP | Synonymous | SAR0924 | *pgi* | AAA | AAG | glucose-6-phosphate isomerase | G |
| K | 365506 | G | A | SNP | Synonymous | SAR0870 |  | CTA | TTA | ABC transporter ATP-binding protein | P |
| K | 470770 | G | T | SNP | Non-synonymous | SAR2150 |  | GGC | GTC | hypothetical protein | S |
| K | 524978 | T | C | SNP | Synonymous | SAR1419 |  | TTA | TTG | putative branched-chain amino acid transporter protein | E |
| K | 526867 | G | A | SNP | Synonymous | SAR1420 |  | GAC | GAT | hypothetical protein | P |
| K | 621294 | C | T | SNP | Premature stop | SAR1965 |  | CGA | TGA | ThiJ/PfpI family protein | R |
| K | 636737 | C | T | SNP | Non-coding |  |  |  |  |  |  |
| K | 719148 | G | A | SNP | Synonymous | SAR2726 |  | AAC | AAT | hypothetical protein | - |
| K | 728171 | C | T | SNP | Non-synonymous | SAR2733 | *secY* | GTT | ATT | preprotein translocase subunit SecY | U |
| K | 739341 | T | C | SNP | Non-synonymous | SAR2743 |  | AAT | GAT | putative capsule synthesis protein | MG |
| K | 745014 | C | A | SNP | Synonymous | SAR2750 | *icaC* | ACC | ACA | intercellular adhesion protein C | G |
| K | 805878 | T | C | SNP | Non-synonymous | SAR2186 |  | GAA | GGA | hypothetical protein | - |
| K | 1024737 | T | C | SNP | Synonymous | SAR2437 |  | GTT | GTC | putative transport protein | G |
| K | 1024737 | T | C | SNP | Non-synonymous | SAR2505 |  | TTA | TCA | putative transport system protein | G |
| K | 1396106 | A | G | SNP | Non-synonymous |  |  | GTT | GCT |  | - |
| K | 1425484 | C | A | SNP | Non-synonymous | SAR1673 | *aroE* | TGG | TGT | shikimate 5-dehydrogenase | E |
| K | 1451734 | G | A | SNP | Non-synonymous | SAR1702 |  | GCA | GTA | putative cysteine desulfurase | E |
| K | 1568002 | C | T | SNP | Non-synonymous | SAR1810 | *fhs* | GCA | ACA | formate--tetrahydrofolate ligase | F |
| K | 1793812 | T | C | SNP | Synonymous | SAR1452 |  | CGT | CGC | putative 5-3 exonuclease | L |
| K | 1818124 | T | C | SNP | Non-synonymous | SAR0753 | *fruA* | TTT | CTT | PTS transport system, fructose-specific IIABC component | G |
| K | 1851204 | A | T | SNP | Non-coding |  |  |  |  |  | - |
| K | 1909470 | A | G | SNP | Non-coding |  |  |  |  |  | - |
| K | 1940981 | C | T | SNP | Non-coding |  |  |  |  |  | - |
| K | 1985954 | A | G | SNP | Synonymous | SAR0453 |  | GAA | GAG | hypothetical protein | S |
| K | 2018937 | A | G | SNP | Non-coding |  |  |  |  |  | - |
| K | 2257979 | A | G | SNP | Non-synonymous | SAR1447 | *ebh* | TAA | CAA | very large surface anchored protein | D |
| K | 2357593 | G | A | SNP | Non-synonymous | SAR1367 | *grlA* | CGT | CAT | DNA topoisomerase IV subunit A | L |
| K | 2462990 | C | T | SNP | Non-synonymous | SAR1948 | *glnQ* | CCT | CTT | glutamine transport ATP-binding protein | E |
| K | 2539653 | T | G | SNP | Non-synonymous | SAR2141 | *ilvB* | TAT | GAT | acetolactate synthase large subunit | EH |
| K | 719527 | A | AA | Indel | Frameshift  No premature stop | SAR2726 |  |  |  | hypothetical protein | - |
| K | 819122 | TT | T | Indel | Non-coding |  |  |  |  |  |  |
| K | 1660967 | A | AA | Indel | Frameshift  Premature stop | SAR1897 |  |  |  | hypothetical protein | - |
| K | 2092742 | AA | A | Indel | Non-coding |  |  |  |  |  |  |
| L | 673054 | T | C | SNP | Non-synonymous | SAR2680 | *ldh2* | GTT | GCT | L-lactate dehydrogenase 2 | C |
| L | 1344440 | C | T | SNP | Non-synonymous | SAR2522 |  | CCC | TCC | putative glycerate kinase | G |
| L | 1581511 | G | A | SNP | Non-synonymous | SAR0339 |  | TCA | TTA | putative acetyltransferase | J |
| L | 1955081 | T | C | SNP | Synonymous | SAR2050 |  | GTT | GTC | hypothetical protein | S |
| L | 2190965 | G | T | SNP | Non-synonymous |  |  | AAG | AAT |  | - |
| L | 2551776 | C | T | SNP | Synonymous |  |  | GAC | GAT |  | - |
| M | 552796 | C | T | SNP | Non-synonymous | SAR0156 | *capF* | ACG | ATG | capsular polysaccharide synthesis enzyme | M |
| M | 680586 | A | G | SNP | Non-coding |  |  |  |  |  |  |
| M | 700440 | G | A | SNP | Non-synonymous | SAR0906 |  | GCT | GTT | hypothetical protein | Q |
| M | 707486 | G | A | SNP | Non-synonymous | SAR0900 |  | ATG | ATA | putative pyridine nucleotide-disulphide oxidoreductase | C |
| M | 1059724 | G | A | SNP | Synonymous | SAR1292 |  | TTG | TTA | FtsK/SpoIIIE family protein | D |
| M | 1358808 | C | T | SNP | Synonymous | SAR1048 | *purD* | GGC | GGT | phosphoribosylamine--glycine ligase | F |
| M | 1661419 | A | G | SNP | Non-synonymous | SAR1745 |  | ATC | ACC | hypothetical protein | R |
| M | 1927366 | T | C | SNP | Non-coding |  |  |  |  |  |  |
| M | 2100132 | A | T | SNP | Non-synonymous | SAR0251 |  | TTG | ATG | putative teichoic acid biosynthesis protein | M |
| M | 2632106 | T | C | SNP | Non-synonymous | SAR1605 | *accB* | GTG | GCG | biotin carboxyl carrier protein of acetyl-CoA carboxylase | I |
| M | 2716297 | A | C | SNP | Non-synonymous | SAR0002 | *dnaN* | GAA | GCA | DNA polymerase III subunit beta | L |
| M | 1529712 | T | TAT | Indel | Non-coding |  |  |  |  |  |  |
| M | 1647855 | G | GG | Indel | Non-coding |  |  |  |  |  |  |
