# Supplementary material for: Within-Host Evolution of Staphylococcus aureus during Asymptomatic Carriage
Source: PLoS One. 2013 May 1;8(5):e61319. doi: 10.1371/journal.pone.0061319 (PMC3641031; doi:10.1371/journal.pone.0061319)
Supplement: Table S2 — Evidence for recombination within and between hosts. (DOC) [file pone.0061319.s003.doc]

**Table S2. Evidence for recombination within and between hosts**

|  | **Genomes** | **Homoplasy detected ^a^** | **Correlation between distance and LD ^b^** | ***p*-value ^c^** |
| --- | --- | --- | --- | --- |
| **Within hosts** | |  |  |  |
|  | A | No | 0.12 | 0.730 |
|  | B | No | -0.01 | 0.393 |
|  | C | No | -0.07 | 0.200 |
|  | D | No | -0.27 | 0.220 |
|  | E | No | - | - |
|  | F | No | -0.03 | 0.350 |
|  | G | No | - | - |
|  | H | No | - | - |
|  | I | No | 0.01 | 0.603 |
|  | J | No | -0.04 | 0.323 |
|  | K | No | 0.01 | 0.566 |
|  | L | No | -0.52 | 0.067 |
|  | M | No | -0.26 | 0.036 |
| **Between hosts** | |  |  |  |
|  | CC22 (A-G) | No | -0.11 | < 0.0001 |
|  | CC30 (H-M) | Yes | -0.12 | < 0.0001 |

^a^ Homoplasies were defined using the four gamete test. ^b^ Linkage disequilibrium (LD) was measured by *r*^2^. Where there were fewer than two SNPs, the correlation could not be calculated. ^c^ Determined by 9,999 random permutations of the physical positions of the SNPs.
