# Supplementary material for: Within-Host Evolution of Staphylococcus aureus during Asymptomatic Carriage
Source: PLoS One. 2013 May 1;8(5):e61319. doi: 10.1371/journal.pone.0061319 (PMC3641031; doi:10.1371/journal.pone.0061319)
Supplement: Table S3 — Evidence of transmission between hosts. (DOC) [file pone.0061319.s004.doc]

**Table S3.** Evidence of transmission between hosts

|  |  |  |  |  |  |  | Slow transmission | | Rapid transmission | |
| --- | --- | --- | --- | --- | --- | --- | --- | --- | --- | --- |
| Transmission pair | | | Observed pairwise differences | % sites uncalled | Recombination detected by ClonalFrame | Corrected pairwise differences | Estimated # transmissions  (95% CI) | | Estimated # transmissions  (95% CI) | |
| **CC22** | | | | | | | | | | |
|  | A | B | 239 | 13.7 | Yes | 141 | 6 | (2,11) | 23 | (14,34) |
|  |  | C | 256 | 13.4 | No | 282 | 12 | (6,19) | 47 | (34,50) |
|  |  | D | 293 | 13.8 | Yes | 194 | 8 | (4,15) | 32 | (21,44) |
|  |  | E | 287 | 13.6 | Yes | 197 | 8 | (4,15) | 32 | (22,45) |
|  |  | F | 302 | 13.9 | Yes | 206 | 9 | (4,15) | 34 | (23,46) |
|  |  | G | 154 | 14.3 | No | 150 | 7 | (3,12) | 25 | (15,36) |
|  | B | C | 544 | 14.0 | Yes | 355 | 15 | (8,23) | 50 | (40,50) |
|  |  | D | 317 | 14.3 | Yes | 226 | 10 | (5,16) | 37 | (27,49) |
|  |  | E | 313 | 14.0 | Yes | 227 | 10 | (5,17) | 37 | (27,49) |
|  |  | F | 325 | 14.3 | Yes | 236 | 10 | (5,17) | 39 | (29,50) |
|  |  | G | 172 | 14.8 | No | 174 | 8 | (3,13) | 29 | (19,41) |
|  | C | D | 1254 | 12.2 | Yes | 602 | 25 | (16,35) | 50 | (46,50) |
|  |  | E | 966 | 13.4 | Yes | 580 | 24 | (16,35) | 50 | (46,50) |
|  |  | F | 1254 | 12.3 | Yes | 609 | 26 | (17,36) | 50 | (46,50) |
|  |  | G | 611 | 14.1 | Yes | 545 | 23 | (15,33) | 50 | (45,50) |
|  | D | E | 68 | 12.5 | No | 76 | 3 | (1,7) | 12 | (6,20) |
|  |  | F | 84 | 10.9 | No | 92 | 4 | (1,8) | 15 | (8,24) |
|  |  | G | 41 | 13.7 | No | 46 | 2 | (1,5) | 7 | (3,14) |
|  | E | F | 78 | 12.6 | No | 87 | 4 | (1,8) | 14 | (7,23) |
|  |  | G | 42 | 13.5 | No | 47 | 2 | (1,5) | 8 | (3,14) |
|  | F | G | 53 | 13.8 | No | 60 | 3 | (1,6) | 10 | (4,17) |
| **CC30** | | | | | | | | | | |
|  | H | I | 589 | 14.5 | Yes | 400 | 17 | (10,25) | 65 | (49,82) |
|  |  | J | 538 | 13.0 | Yes | 427 | 18 | (10,26) | 69 | (52,87) |
|  |  | K | 384 | 15.3 | No | 425 | 18 | (10,26) | 69 | (52,87) |
|  |  | L | 394 | 14.8 | Yes | 297 | 12 | (7,20) | 48 | (34,63) |
|  |  | M | 786 | 13.4 | Yes | 484 | 20 | (12,29) | 78 | (61,98) |
|  | I | J | 365 | 13.8 | No | 382 | 16 | (9,24) | 62 | (46,79) |
|  |  | K | 421 | 15.0 | Yes | 392 | 16 | (9,24) | 63 | (48,81) |
|  |  | L | 370 | 14.2 | Yes | 254 | 11 | (5,17) | 41 | (28,55) |
|  |  | M | 498 | 14.2 | Yes | 427 | 18 | (10,26) | 69 | (52,87) |
|  | J | K | 389 | 15.1 | No | 428 | 18 | (10,26) | 69 | (52,87) |
|  |  | L | 237 | 14.1 | No | 263 | 11 | (6,18) | 42 | (30,57) |
|  |  | M | 645 | 12.4 | Yes | 472 | 19 | (12,29) | 76 | (59,95) |
|  | K | L | 268 | 15.4 | No | 297 | 12 | (7,20) | 48 | (34,63) |
|  |  | M | 423 | 15.8 | No | 472 | 19 | (12,29) | 76 | (59,95) |
|  | L | M | 211 | 14.6 | Yes | 204 | 9 | (4,15) | 33 | (22,46) |
