# Supplementary material for: Within-Host Evolution of Staphylococcus aureus during Asymptomatic Carriage
Source: PLoS One. 2013 May 1;8(5):e61319. doi: 10.1371/journal.pone.0061319 (PMC3641031; doi:10.1371/journal.pone.0061319)
Supplement: Text S1 — Adjusting Observed SNP Differences for Call Rates and Recombination. (DOC) [file pone.0061319.s005.doc]

**W****ithin-host evolution of *Staphylococcus aureus* during asymptomatic carriage**

Tanya Golubchik, Elizabeth M. Batty and colleagues

## Text S1: Adjusting Observed SNP Differences for Call Rates and Recombination

To estimate the number of transmission events separating a pair of *S. aureus* genomes isolated from two hosts, we analyzed the number of pairwise SNP differences using the metapopulation model described in the Methods. However, to obtain reliable estimates we first adjusted the observed SNP differences between pairs of genomes to correct for differences in

1. the proportion of sites filtered out by our SNP calling pipeline, and
2. the effect of recombination.

This was necessary, first, because the effect of filtering is to reduce the number of sites called, and therefore to reduce the number of SNP differences detected compared to what would be observed if all sites were correctly called. Second, homologous recombination can result in the appearance of clusters of SNP differences in the genome, therefore increasing the expected number of SNP differences detected compared to what would be observed if divergence was driven exclusively by point mutations.

## Homologous recombination

To adjust the number of pairwise SNP differences (the *pairwise difference*s) for the effect of homologous recombination, we used the model underlying ClonalFrame [1]. In the ClonalFrame model, mutation occurs at rate *θ_s_* per nucleotide and recombination is initiated at rate *ρ_s_* per nucleotide. When recombination occurs, the length of homologous DNA imported is assumed to follow a geometric distribution with mean *δ* nucleotides. The nucleotide divergence of the imported DNA is specified by the parameter *ν*.

We wished to estimate the number of pairwise differences attributable to mutation. To do so, we first estimated *T*, the length of the branch separating the two genomes in a two-taxa tree, measured in units of the expected number of point mutations per nucleotide. We then defined the adjusted pairwise difference to be *TG*, where *G* is the length of the genome.

To estimate *T* in a computationally efficient manner, we used the ClonalFrame model, which can be thought of as a hidden Markov model (HMM, see, e.g. [2]) when there are only two genomes. The hidden state of the HMM records whether each nucleotide was subject to recombination or not on the branch connecting the two genomes. Nucleotides unaffected by recombination are said to be *unimported* and nucleotides subject to recombination are said to be *imported* [1]. Based on the ClonalFrame model, we defined the following transition probability matrix for the hidden variable between adjacent sites, *H_i_* and *H_i_*_+1_:

The emission probability defines the likelihood for the observed data conditional on the underlying hidden variable. At each nucleotide, *i*, we summarized the observed data, *D_i_*, as either the *same* or *different*, depending on whether the two genomes matched or not. If the site was not called in either or both genomes, we defined it to be the *same*. In the next section we correct for this simplification, which was computationally convenient. Following the ClonalFrame model, we defined the following emission probabilities for the data at nucleotide *i*:

We used the forward algorithm [2] to calculate the likelihood . We estimated *T* using maximum likelihood, implemented as a unidirectional optimization routine, taking as the values of the auxiliary parameters the point estimates from the full ClonalFrame analyses, which were performed separately for the CC22 and CC30 genomes.

## Call rates

To adjust the pairwise differences for the call rate between each pair of genomes (defined as the proportion of the genome not filtered by the SNP calling pipeline), we calculated the proportion of nucleotides, *p_N_*, that were not called in either or both genomes. We took the final adjusted number of pairwise SNP differences to be , rounded to the nearest integer.

## References

## Didelot X, Falush D (2007) Inference of bacterial microevolution using multilocus sequence data. Genetics 175: 1251-1266.

## Durbin R, Eddy SR, Krogh A, Mitchison G (1998) Biological Sequence Analysis: Probabilistic Models of Proteins and Nucleic Acids. Cambridge: Cambridge University Press.
